# Effect of Lure Combination on Fruit Fly Surveillance Sensitivity

**DOI:** 10.1038/s41598-018-37487-6

**Published:** 2019-02-25

**Authors:** Lloyd D. Stringer, Rajendra Soopaya, Ruth C. Butler, Roger I. Vargas, Steven K. Souder, Andrew J. Jessup, Bill Woods, Peter J. Cook, David Maxwell Suckling

**Affiliations:** 1 The New Zealand Institute for Plant & Food Research Limited, Private Bag 4704, Christchurch, 8140 New Zealand; 2 Better Border Biosecurity, Auckland, New Zealand; 3 Centre for Biodiversity and Biosecurity, School of Biological Sciences, University of Auckland, Auckland, New Zealand; 4 Department of Primary Industries and Regional Development, South Perth WA, Australia; 5 Plant Biosecurity Cooperative Research Centre, Bruce, Canberra, ACT, Australia; 6 Daniel K. Inouye, U.S. Pacific Basin Agricultural Research Center, Agricultural Research Service, United States Department of Agriculture, Hilo, HI USA; 7 NSW Department of Primary Industries, Central Coast Primary Industries Centre, Ourimbah, NSW Australia; 8 Present Address: Janren Consulting Pty Ltd, Bateau Bay, NSW Australia; 9 Farma Tech International Corporation, North Bend, WA USA

## Abstract

Surveillance for invading insect pests is costly and the trapper usually finds the traps empty of the target pest. Since the successful establishment of new pests is an uncommon event, multiple lures placed into one trap might increase the efficiency of the surveillance system. We investigated the effect of the combination of the Tephritidae male lures – trimedlure, cuelure, raspberry ketone and methyl eugenol – on catch of *Ceratitis capitata*, *Zeugodacus cucurbitae*, *Bactrocera tryoni*, *B*. *dorsalis*, *B*. *aquilonis* and *B*. *tenuifascia* in Australia and the USA (not all species are present in each country). The increase in trap density required to offset any reduction in catch due to the presence of lures for other Tephritidae was estimated. The effect of increasing trap density to maintain surveillance sensitivity was modelled for a hypothetical population of *B*. *tryoni* males, where the effective sampling area of cuelure traps for this species has been estimated. The 3-way combination significantly reduced the catch of the methyl eugenol-responsive *B*. *dorsalis*. Unexpectedly, we found that trimedlure-baited traps that contained methyl eugenol had ×3.1 lower catch of *C*. *capitata* than in trimedlure-only-baited traps in Australia, but not in Hawaii where no difference in catch was observed, we cannot satisfactorily explain this result. Based on the data presented here and from previous research, combinations of some male lures for the early detection of tephritid flies appear compatible and where there is any reduction in surveillance sensitivity observed, this can be offset by increasing the density of traps in the area.

## Introduction

Surveillance for biosecurity pests is conducted to detect new species incursions as soon as possible after incursion, in order to support trade while mitigating the risk of pest establishment. A reduction in the sensitivity of a surveillance detection grid could lead to a delayed detection of the target species, which could increase the time taken to eradicate it, and consequently, the cost required for an eradication programme^1–3^. One of the more costly aspects of a surveillance grid is trap servicing^4^. In cases where trapping grids are placed for the early detection of new species incursions, traps are likely to be empty the majority of the time. While finding an empty trap is a desired outcome, as it indicates that the pest is unlikely to be present, it would be beneficial from a cost perspective if the surveillance system was to target more than one species at a time.

Insect trapping systems often use odours such as pheromones or host plant volatiles to attract insects to a trap. Odours are a powerful monitoring tool for the early detection of a species and population monitoring^5^. Lure combinations offer the prospect of greater surveillance effort being achieved for only a small increase in the cost for the additional lures and no significant increase in labour costs, making such efficiency gains attractive. Recent work in Australia and the USA has sought to trap several species of fruit fly (Diptera: Tephritidae) using various lure combinations^6–10^. There are other examples of combinations of lures for trapping moths^11,12^, beetles^13,14^ and species from other insect orders^15^. However, some odours contained in a single trap may not be compatible, thus reduce the catch of the target^11^. For example, related sympatric species *Archips argyrospilus* and *A*. *mortuanus* use the same sex pheromone compounds but in different ratios^16^. It is unlikely that both pheromone blends offered together would attract both species as effectively as their respective blends presented singly. Further, odour incompatibility may occur between species that are not as closely related^17^. For the attraction of male Tephritidae using male attractants, it has been observed that the presence of cuelure reduces the catch of the methyl eugenol responsive *B*. *dorsalis*^18^, but this reduction can be overcome by increasing the amount of methyl eugenol released when presented together with cuelure^10^.

The sensitivity of a trapping system is influenced by the trap and lure combination as well as the configuration of the trap system^19^. The efficacy of the system can be predicted by estimating the probability of catch of an insect from a population as a function of distance from a trap such as, but not limited to, the effective sample area (ESA)^20,21^ or the effective attraction radius^22–24^. The ESA multiplied by the density of traps in the target area then gives the amount of trap cover by a surveillance system, up to a maximum of 100% coverage when there is ESA overlap. While a surveillance system may have 100% coverage of an area, this does not mean that the system will catch 100% of the target insects present^25^. Trap sensitivity is dictated by the effectiveness of the lure. For example, highly attractive lures require fewer traps per area than less attractive lures, which require a higher density of traps baited with the lures to have a comparable surveillance efficacy^23,26–31^.

We trialled the combination of multiple male lures: raspberry ketone, cuelure, trimedlure and methyl eugenol, for use in fruit fly (Diptera: Tephritidae) surveillance traps. We estimated the change in efficacy from a single lure trapping system for a particular species (*Bactrocera tryoni*) in the presence of additional lures that target other species. Any reduction in catch may be a result of a change in the behaviour of the target insect. The additional odours may reduce the attraction of the target by reducing release rates in the mixtures through chemical interactions when the compounds are combined on the same substrate. Further, attraction may be reduced because the additional odours act as antagonists, odours that are perceived by the target that reduce behavioural attraction as has been observed in moths reacting to sex pheromones containing compounds of related species^32^. This change reduces the effective sampling area of a trap. We estimate what a potential change in grid sensitivity for a target species, because of the presence of a lure for an additional species, may mean for the probability of detection for that target. When there has been a reduction in sensitivity in the grid due to the additional lures, we estimate whether the original sensitivity of the surveillance system can be regained by increasing the density of traps of the less sensitive multi-lure system.

## Results

### Fruit fly lure combinations

#### Hawaii and New South Wales (Australia)

**Figure 1:**
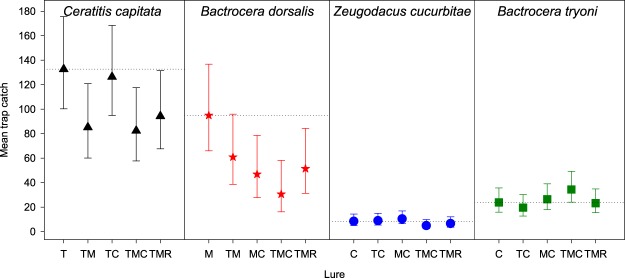
Mean catches of three species of fruit fly in Hawaii (catch/24 h), and one in Australia (right panel; catch/37 days) in the presence of attractants for different fruit fly species. C = cuelure, M = methyl eugenol, T = trimedlure, R = raspberry ketone. Dotted horizontal lines in each panel are at the means for the single-component lure. Error bars show 95% confidence limits for the means.

The catch per trap of *C*. *capitata* males did not vary significantly between the different lure types (F = 1.26_(4,45)_, P = 0.299). Catch of male *C*. *capitata* in traps containing the addition of methyl eugenol was 62–64% of the catch in traps that contained trimedlure alone. Catches of both *Z*. *cucurbitae* males (F = 0.62_(4,45)_, P = 0.649) and *B*. *tryoni* (Χ^2^ = 7.49, d.f. = 4, P = 0.112) did not differ significantly for the lure combinations tested. There was a significant reduction in catch of *B*. *dorsalis* only in the combined lure traps containing trimedlure, methyl eugenol and cuelure (TMC) (F = 0.62_(1,45)_, P = 0.012); catch in the combination lure was 32% of the catch in the methyl eugenol-only-baited traps (Fig. 1).

#### Western Australia trial 1

**Figure 2:**
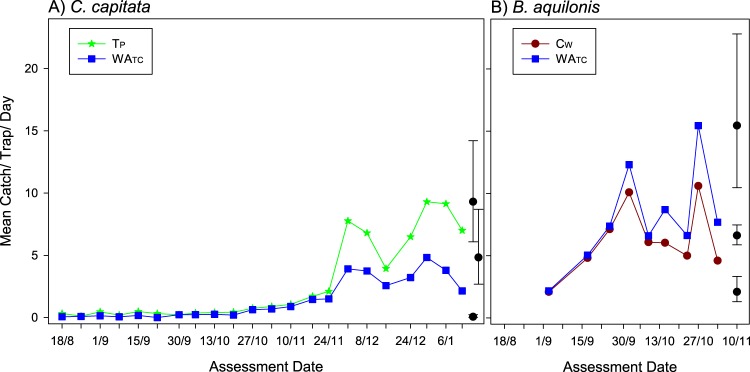
Mean trap catch at each assessment date for each of two lures for *Ceratitis capitata* and *Bactrocera aquilonis* male fruit fly in trial 1 in Western Australia. Tp = 3 g trimedlure plug, Cw = 1 g cuelure on a cotton dental wick, and WATC trimedlure 3 g plug + cuelure 1 g wafer. Error bars show 95% confidence limits: for clarity, these are given for just the largest, smallest and a mid-range mean from within each plot, redrawn to the side.

The mean catch per trap per day for the two *C*. *capitata* treatments for each assessment period (time between trap checks) is shown in Fig. 2. Catch was very low from the commencement of trapping in mid-August until mid-October, with catches in almost all traps remaining below five flies per trap per day before this date. At the end of October, mean trap catches increased steadily for both lure types until after 24 November, when catches increased dramatically. After this date, there was greater than 4-fold increase in catch for the next assessment for the trimedlure, and a more than 3-fold increase in catch for the combination lure. Catches remained relatively high until the final assessment.

The combination lure caught just over half the number, mean 215.2 (confidence limits: 122.2, 379), of *C*. *capitata* that were caught with trimedlure alone, mean 418.8 (confidence limits: 279.1, 628.3). However, this difference was not statistically significant (F = 5.13_(1,8)_, P = 0.053), largely because of the small number of replicates (only 10 traps), and the generally high variation between catches.

For *B*. *aquilonis* (Fig. 2), mean catch summed over all assessments was about 25% greater with the combination lure (498 per trap [confidence limits 410.7, 603.8]) than with cuelure alone (403 per trap [confidence limits 325.3, 499.2]), although this effect was not significant (F = 2.89_(1,8),_ P = 0.128).

#### Western Australia trial 2

**Figure 3:**
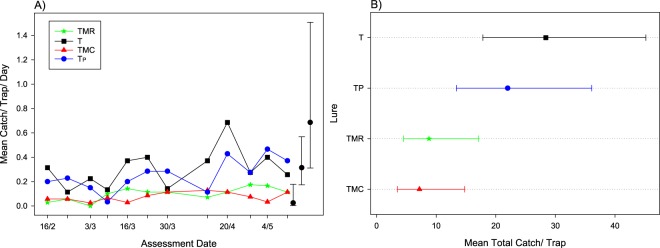
Mean *Ceratitis capitata* male trap catch per day for each assessment period for four lures (**A**), and mean trap catch (**B**) for the entire trial with lures ordered by their means in trial 2 in Western Australia. Error bars (**A**) show 95% confidence limits: for clarity, these are given for just the largest, smallest and a mid-range mean from within each plot, redrawn to the side. For (**B**), error bars are 95% confidence limits. Letters in the legend represent: C = cuelure, M = methyl eugenol, T = trimedlure, R = raspberry ketone and Tp = 3 g trimedlure plug.

In the trials testing additional Farma Tech combination lures against the standard lures for surveillance, the mean *C*. *capitata* trap catch per day for the four treatments at each assessment was low for the entire trial, with catches in all traps remaining below 2 flies per trap per day (Fig. 3). There were differences in fly catch between replicates, indicating possible fly hotspots, so this was adjusted for in the final analysis by including replicate as a random effect. Even after adjusting for replicates, there was high over-dispersion, so this was allowed for in the final Poisson-gamma HGLM used.

The two trimedlure-only lures, the wafer and plug (T, Tp) caught on average three times as many *C*. *capitata* (28 and 22 per trap respectively (confidence limits: 17.8, 45.2 and 13.4, 36.1 respectively); mean = 25; T vs TP [F = 1.40_(1,13)_, P = 0.258]) over the trial than the two wafer combination lures (TMR, TMC with 9 and 7 per trap respectively (confidence limits: 4.5, 17.1 and 3.5, 14.8 respectively); mean = 8; TMR vs TMC [F = 0.03_(1,13)_, P = 0.865]; T or Tp vs TMR or TMC [F = 9.45–17.52_(1,13)_, 0.001 < P < 0.005]) (Fig. 3).

**Figure 4:**
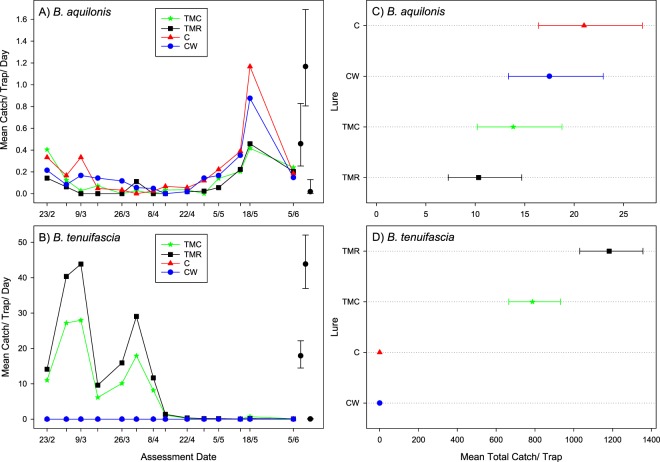
Mean catch per trap per day of *Bactrocera aquilonis* (**A**) and *Bactrocera tenuifascia* (**B**) males at each assessment for each of the four lures in trial 2 in Western Australia. Mean trap catch (totalled over assessments) for *Bactrocera aquilonis* (**C**) and *Bactrocera tenuifascia* (**D**) males, for four lures, with lures ordered by their means. Error bars show 95% confidence limits: clarity, these are given for just the largest, a small, and a mid-range mean from within the plot, redrawn to the side. Note that an upper confidence limit for a mean of 0 cannot easily be obtained, and so is not shown. Letters in the legend represent: C = cuelure, M = methyl eugenol, T = trimedlure, R = raspberry ketone and Cw = 1 g cuelure on a cotton dental wick.

The mean catch per trap per day in the four treatments for *B*. *aquilonis* was quite low for the trial (Fig. 4A). Changes in *B aquilonis* catch over time were quite similar for all four lures, with a peak in catch in all treatments at the second to last assessment (18 May 2015). Catch per trap per day of *B*. *tenuifascia* was initially high then dropped to near zero for the remainder of the trial and no flies were caught with the C or CW lures (Fig. 4B).

For *B*. *aquilonis* catches totalled over all assessments, there were no strong spatial trends across the trial, so no adjustments for such trends were made in the final analysis. There was moderate over-dispersion, so this was allowed for in the analysis. There were significant differences in mean total catch between the treatments (F = 4.66_(3,20)_, P = 0.013 for an overall test): catches for the C (mean, 21.0 per trap [confidence limits: 16.4, 26.9]) and CW (mean, 17.5 per trap [confidence limits: 13.3, 22.9]) lures were the highest, significantly larger than those for TMR (mean 10.3 per trap [confidence limits: 7.3, 14.7]) in both cases (t = 3.43, d.f. = 20, P = 0.003 and t = 2.47, d.f. = 20, P = 0.023 respectively), but significantly greater than TMC (mean 13.8 per trap [confidence limits: 10.2, 18.8]) only for C (CW: t = 1.20, d.f. = 20, P = 0.243; C: t = 2.24, d.f. = 20, P = 0.038). Differences between CW and C, and TMR and TMC were not significant (t = 1.04, d.f. = 20, P = 0.312 and t = 1.31, d.f. = 20, P = 0.207 respectively) (Fig. 4C).

For *B*. *tenuifascia*, there were between replicate differences. However, since none of this species was caught in the C and CW traps, adjustment for such a difference can be unreliable. Therefore, as for *B*. *aquilonis*, no adjustments for such trends were made in the final analysis, and the substantial over-dispersion was allowed for in the analysis. There were significant differences in catch between the lures (F = 182.6_(3,20)_, P < 0.001 for an overall test), which related primarily to there being no catch with the C and CW lures, but a large catch with both of the other lures. However, the catch with TMR was about 1.5x that with TMC (t = 82.6, d.f. = 20, P < 0.001), at 1182 per trap (confidence limits: 1031, 1357) compared with 787 per trap (confidence limits: 665, 931) (Fig. 4D).

### Surveillance sensitivity

**Figure 5:**
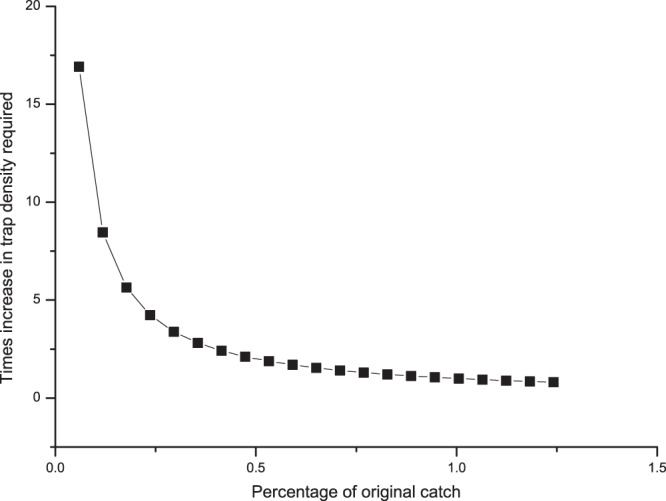
The modelled number of traps required to maintain trap grid sensitivity as a function of percentage change in trap sensitivity.

The predicted multiple of increase in the density of traps required when using traps with a combination of lures that are less sensitive than a single lure surveillance system was modelled (Fig. 5). The model behaved as expected and the predicted number of male *B*. *tryoni* trapped in a surveillance grid that used cuelure only was equal to the number predicted to be caught in a less sensitive surveillance grid that had a greater density of traps to offset catch reduction. By using eqn. 2 (see methods), it is predicted that to maintain a similar probability of detecting *B*. *dorsalis* when using the TMC combination, which had 32.2% of the catch of methyl eugenol alone, 3.1 times the density of TMC traps would be required to maintain the sensitivity of a methyl eugenol only trapping grid (32.2 × 3.1 = 100). For example, if this was translated to the New Zealand fruit fly surveillance trapping grid, this would require a change from methyl eugenol traps placed 1200 m apart, 0.007 traps/ha^33^, to TMC combination lure traps placed 600 m apart, 0.027 traps/ha. The surveillance grid for both trimedlure and cuelure responsive species in New Zealand has a closer trap spacing 400 m apart, 0.063 traps/ha^33^. If the combination lure was used in traps for surveillance for *B*. *dorsalis*, as well as for the trimedlure- and cuelure-responsive species, placed 400 m apart, grid sensitivity for *B*. *dorsalis* would still be as good as, if not better than, *B*. *dorsalis*-lure-only baited traps placed 1200 m apart. The percentage reduction in trapping results for *C*. *capitata* in Western Australia with the combination lure was similar to that of *B*. *dorsalis* in Hawaii; thus the calculated required increase in density was also similar (×3.1). This was derived by using eqn. 2, a mean of eight were trapped in the TMC combination trap and 25 in the single trimedlure trap, 8/25 = 0.32, so catch in the combination trap was 32% of catch in the single lure trap. The complement of this is 1/0.32 = 3.1. Using a similar process for *B*. *aquilonis*, an increase of ×1.7 the traps would be required to maintain the expected sensitivity of a grid of single cuelure traps if a combination of TMR was used.

## Discussion

Trapping of the cuelure-responsive species *Z*. *cucurbitae*, and *B*. *tryoni*, and of the trimedlure-responsive species, *C*. *capitata*, in the presence of each other’s lures in the same trap did not significantly reduce the efficacy of the respective traps in the Hawaiian trial. These results corroborate earlier results from previous research^8–10^.

The catch of *B*. *dorsalis* attracted to methyl eugenol was significantly reduced in traps that contained trimedlure, cuelure and methyl eugenol together. This reduction could be offset by either increasing the amount of methyl eugenol on the combination lure^10^ or by increasing the density of traps used for the early detection of *B*. *dorsalis* (e.g.^34^).

Catch of *C*. *capitata* was greater in the standard 3-g trimedlure plug-alone trap than in the plug plus cuelure combination in Western Australia. Based on results in Hawaii and in the literature^7–10,35^ we did not expect to see an effect of cuelure on *C*. *capitata* catch. The lures were not replaced during the 20-week trial (Fig. 2A) and it is possible that the effect of lower catch in the combination lure could be related to the ratio of trimedlure to cuelure changing over the course of the study, which was longer than the trial by Vargas *et al*.^35^ where lures were aged for eight weeks. Ratios of lures in a combination trap have been shown to play an important role on the catch of different fruit fly species^10^. Trimedlure appears to be the most volatile of the compounds tested here, followed by methyl eugenol and cuelure^35–37^ and it likely that the trimedlure: cuelure ratio changed from the beginning to the end of the trial. As the attractants used in Western Australia trial 1 differed to those used in the Hawaiian trial, we re-ran the trial using some of the same lures as used in Hawaii with the expectation that the catches of *C*. *capitata* in traps baited with trimedlure alone and with the full combination in Western Australia trial 2 may be slightly but not significantly reduced. However, again we saw an effect and found that the combination lure traps caught only a third of the numbers caught with trimedlure-only traps (Fig. 3). Further, catch was consistently higher in the trimedlure-alone traps through time, thus rejecting our hypothesis that lure age had influenced results in Western Australia. Previous trials^8,9,35^ and the trial here have shown that the presence of methyl eugenol can reduce catch of *C*. *capitata*, but this reduction is not expected to be significant. It is unlikely that methyl eugenol is naturally abundant in citrus orchards in Australia where the trials were conducted, as the only time methyl eugenol has been reported from citrus is after the application of an abscission compound that was assessed to help with fruit harvest^38^. This is the first time the combination lure has been trialled on *C*. *capitata* outside of Hawaii. It appears that geographic, climate and/or habitat differences have affected the combination lure to lead to the reduction in *C*. *capitata* catch in Western Australia. However, because geographic, climate and habitat differences between the two sites were not explicitly tested, we thought that further comparisons between a 1-day trial (Hawaii) and a 91-day trial (Western Australia trial 2) would be inappropriate and could be misleading. At this time, we cannot conclude as to why we have observed this difference in *C*. *capitata* catch between the two sites in the presence of cuelure and methyl eugenol.

It appeared that the combination of trimedlure and methyl eugenol negatively affected the catch of *B*. *aquilonis*. Catches of *B*. *aquilonis* to both the cuelure-alone lures and the cuelure plus trimedlure traps were similar in Western Australia trial 1. Since catch was not different in cuelure-alone and cuelure plus trimedlure traps, it is likely that the presence of methyl eugenol affected catch. *B*. *tenuifascia* was not trapped in cuelure-only baited traps, but was attracted to the combination trap containing all lures. Catch was ×1.5 greater in the TMR traps than in the TMC traps, suggesting that cuelure has a negative effect on catch. It has been shown that sexually mature virgin females of both *B*. *tenuifascia* and *B*. *aquilonis* are attracted to the male lures methyl eugenol and cuelure, respectively^39^.

The approach we used here to estimate the ratio change in catch to predict the increase in trap density required, offers a system that is not reliant on prior knowledge of the probability of trapping an insect with distance (ESA). But it does need knowledge of the relative effect on catch of the target species with and without additional lure to estimate the density of less-sensitive traps required to maintain the relative surveillance efficacy of the original lure system in a known area. This estimate was tested against the ESA of a cuelure trapping system for *B*. *tryoni*^40^. The ESA value is developed from spatial trapping data such as mark-release-recapture trials where the number of and distance from the traps of the released insects are known. ESAs have not been developed for many species and can differ for the same species depending on lure and trap combinations. One potential pitfall of this approach is that it does not assume any overlap in attraction area so that the traps are not competing with each other for insects^41^. As the density of traps increase, so does the probability that the attraction area of multiple traps will begin to overlap. However, as long as the insect does not suffer from sensory fatigue^42^, but see^43^, the increase of relative trap density improves the likelihood of trapping an attracted insect. But where traps are less sensitive, this effect of competing traps is less likely to be an issue.

The reduced catch of *B*. *dorsalis* in traps that contained trimedlure, methyl eugenol and cuelure (TMC) represented a significant reduction in trap catch which is probably related to a reduction in trap sensitivity. However, because the catch of the other two species was not significantly affected, in the New Zealand trapping system example used here, it is predicted that the probability of *B*. *dorsalis* being trapped with 400 m between traps would be as good if not better than the current 1200m- methyl eugenol-only system. Of concern is the reduction of *C*. *capitata* catch in multi-lured traps in Western Australia, as this would suggest that combination traps should be placed 225 m apart, or 3.1× the density of traps/ha, to maintain the trap cover achieved by the current trimedlure system.

The benefits of combining lures into a single trap are that fewer traps need to be checked. A possible negative effect of combining lures is that a higher density of traps might be required. In the example here, to maintain trap grid sensitivity for *B*. *dorsalis* in a combination-lure trap, more methyl eugenol lures would be required for surveillance, but since the traps that contain trimedlure or cuelure are currently 400 m apart, closer than needed to offset the reduction, the only increase in cost would be the additional lures. If the reduction in catch of *C*. *capitata* in Western Australia was due to the presence of methyl eugenol or cuelure then there is the risk that a combination lure used in a grid of traps placed 400 m apart for the early detection of fruit flies would detect *C*. *capitata* later than a single lure grid. This is because the sensitivity of the trap for *C*. *capitata* is reduced and it was estimated that to maintain grid sensitivity, a combination lure surveillance grid would need traps to be placed 225 m apart. It appears that the reduction in *C*. *capitata* catch due to the presence of additional lures may differ between geographic regions. Post detection, if sensitivity of the trapping system is reduced because of the presence of multiple lures, sensitivity could be increased by placing out the single lure for the target species only. While the probability of fruit fly eradication is high under most scenarios, primarily due to the large numbers of tools available^44^, the risks of late detection are that the population would be larger by the time it was detected, and the probability of accidental transportation elsewhere in a country prior to detection would be greater. This would lead to a prolonged, thus, expensive eradication.

The potential options for surveillance are to: 1. Keep all three lures separate and trap for each species separately; 2. Combine trimedlure and cuelure into individual traps placed at 400 m spacing and keep methyl eugenol in a separate trap spaced 1200 m apart; 3. Combine all lures into one trap, placing these 400 m apart. Based on the results here it appears that the recommendations would change depending on the area that trapping was conducted. In Hawaii, all three lures could be combined, in Western Australia all three lures could be combined but trap density would need to be increased for the early detection of *C*. *capitata*. While an increase in trap density will cost more to service, this cost may be offset by no longer needing separate traps to survey for the methyl eugenol- nor cuelure-responsive species. We do not know whether option 2 is viable as this was not tested in Western Australia. Many countries maintain a trapping grid for the early detection of fruit flies. If those countries are considering on combining lures but do not have any fruit flies present to test the effects of combination on catch, these results are not easy to interpret. Decisions will have to weigh the risks posed to each country. In this and previous trials, the cuelure/raspberry ketone responsive species have not been negatively affected by the presence of trimedlure nor methyl eugenol. However, methyl eugenol –responsive *B*. *dorsalis* catch has been reduced in the presence of other lures, but this reduction in catch (i.e. grid sensitivity) would be offset with the higher density of traps that is often used for cuelure or trimedlure responsive species. The catch of *C*. *capitata* provides the most difficulty for interpretation as this is the first time a reduction in catch has been observed due to the presence of additional lures. We cannot determine whether this is from the presence of cuelure/raspberry ketone or methyl eugenol or both. More work is required to determine why this difference has been observed in Western Australia but not in Hawaii. Comparisons at various locations that have species such as, *C*. *capitata* in Europe^45^ or southern Africa that have both *C*. *capitata*^46^ and *B*. *dorsalis*^47^, coupled with analyses of environmental conditions could help elucidate the differences recorded here.

Large numbers of insects are making their way around the globe and these movements do not appear to be slowing down^44,48^. If climate changes as predicted, new areas may become more climatically suitable for new species^49^. Combining lures for the early detection of a new species offers the ability to maintain surveillance for current threats and for a small increase in cost mainly of lures. Any reduction in trap sensitivity due to the presence of additional lures could be offset by increasing the density of traps.

## Methods

### Fruit fly lure combinations

Combinations of standard commercially available and novel lures were trialled to assess any impact on catch relative to catch with a single-component lure. Trials were carried out in Hawaii, USA, and in New South Wales and Western Australia, Australia. Lures comprised various combinations of raspberry ketone (R), cuelure (C), trimedlure (T) and methyl eugenol (M) (Table 1).

**Table 1:** Compounds used (grams of active ingredient [a.i.]) as well as percentage of a.i. by weight of commercially prepared lures when combined with other compounds* for Tephritidae trapping trials carried out in Hawaii, New South Wales (NSW), and Western Australia (WA) Australia.

Lure	Code	Grams (a.i.)	*Percentage a.i. of compounds by weight of commercially prepared lures when combined with other compounds	Trial sites
Trimedlure wafer*	T	3.5	15.3	Hawaii, NSW and WA
Methyl eugenol wafer*	M	5.5	23.8	Hawaii, NSW and WA
Cuelure wafer*	C	2.0	8.6	Hawaii, NSW and WA
Raspberry ketone wafer*	R	2.0	8.6	Hawaii, NSW and WA
Trimedlure plug	Tp	3.0	—	WA only
Cuelure on cotton wick	Cw	1.0	—	WA only
Trimedlure plug+ cuelure wafer	WA^TC^	3.0 1.0	—	WA only

In Hawaii, the species targeted were Mediterranean fruit fly, *Ceratitis capitata*, attracted to trimedlure; oriental fruit fly, *Bactrocera dorsalis*, attracted to methyl eugenol; and melon fly, *Zeugodacus cucurbitae*, attracted to cuelure and raspberry ketone. In New South Wales, Queensland fruit fly, *B*. *tryoni*, attracted to cuelure and raspberry ketone was targeted, and in Western Australia, *C*. *capitata*, attracted to trimedlure and the Northern Territory fruit fly, *B*. *aquilonis*, attracted to cuelure (a species closely related to *B*. *tryoni*) were targeted^50^. In addition, *B*. *tenuifascia* was attracted in large numbers to methyl eugenol in Western Australia, so the effect of lure combinations was assessed.

Farma Tech International Corp. (North Bend, WA, USA) (www.farmatech.com) fruit fly lures were formulated on to a wafer (polymer matrix) 5.1 × 7.6 × 0.6 cm (2″ × 3″ × 1/4″) at +12%. Lure loadings (+10%) were 5.5 g for methyl eugenol, 2 g for cuelure or raspberry ketone and 3.5 g for trimedlure, the same rates used in trials by Vargas *et al*.^8^. Comparisons between captures of pest fruit fly species associated with the three test sites (i.e. Hawaii, New South Wales and Western Australia) were made using single, double and triple combinations of the lures: trimedlure, methyl eugenol and cuelure. Raspberry ketone was only used in the triple combination. All lures were fitted into Lynfield traps (Cowley 1990). Gloves were changed between distributing the different lure treatments to prevent possible contamination. Dichlorvos-impregnated strips (DDVP strips), Hercon Vaportape II in Hawaii and Killmaster® pest strips in Australia were added to traps to kill flies entering the traps. Traps were placed 20 m apart. As this trial assessed the relative difference in catch between treatments, any reduction in trap catch due to competition between traps was not expected to influence the relative results.

### Hawaiian trial

In Hawaii, trials were conducted for 24 h. This was to simulate areas of lower capture and to prevent traps from overflowing. Trials were conducted near Numila, Kauai Island, HI, at a large (c. 1,400 ha) commercial coffee [*Coffea arabica* L. ‘Arabica’ (Rubiaceae)] plantation (lat. 21.910, long.−159.548), at an average elevation of 125 m, where *Ceratitis capitata*, *Bactrocera dorsalis* and *Zeugodacus cucurbitae* co-occur. Field attraction experiments were conducted on 14–15 November 2013. Traps were hung in coffee trees apart using a randomized complete block design with ten replicates of each treatment (Table 1). The DDVP strips were aged for two days before the trial to reduce any repellent effects.

### New South Wales trial

The effects of trimedlure and methyl eugenol on cuelure- and raspberry ketone- responsive *Bactrocera tryoni* were tested in Somersby, Central Coast of New South Wales (lat. −33.367, long. 151.305). Five replicates of the five treatments were placed out in a mixed citrus orchard in a randomized complete block design. Traps were placed out at 1130 h and were initially operated for 24 h. After 24 h, very few insects were trapped and many zeros were recorded. Traps were operated for a further 36 days (26 February 2014 until 30 March 2014). The DDVP was aged as above.

### Western Australian trials

Various combinations of lures were tested in Western Australia. The combination of trimedlure and cuelure was tested for the trimedlure-responsive *C*. *capitata* in an orange *Citrus* × *sinensis* orchard in the West Swan area (lat. −31.833, long. 116.000) near Perth (Western Australia trial 1), and the cuelure-responsive *B*. *aquilonis* in a grapefruit *Citrus* × *paradisi* orchard in Kununura (lat. −15.779, long. 128.742) (Western Australia trial 2). Lures were not replaced during the trials.

### Western Australia trial 1

The initial trial compared the Farma Tech lures to current standard lures used in Western Australia for *C*. *capitata*, a 3-g trimedlure plug, and for *B*. *aquilonis*, 1 g of cuelure on a cotton dental wick. Five replicates of each treatment were carried out, laid out in a rectangular array of 5 × 2 traps in a systematic design. Because of an initial low population, *C*. *capitata* traps were checked weekly and operated from 8 August 2014 until 15 January 2015. Trapping for *B*. *aquilonis* occurred from 27 August 2014 until 31 November 2014. Traps were checked every 2 weeks.

### Western Australia trial 2

In the second *C*. *capitata* trial the Farma tech lures, T, TMR and TMC combinations were tested as well as current standard trimedlure plug (Tp). The Farma Tech lure loadings were as from Hawaii above and the trimedlure plug (Tp) as in Table 1. Pest strips were added as above. Five replicates of each treatment were used, laid out in a randomized block design. Traps were out from 9 February 2015 until 11 May 2015. Traps were serviced weekly.

The lure combinations tested for *B*. *aquilonis* were the Farma Tech lure, C, TMR and TMC, as well as the standard cuelure-loaded dental wick (Cw). The Farma Tech lure loadings were as above and the cuelure on a cotton dental wick (Cw) as in Table 1. Pest strips were added as above. Six replicates of the lures were used, laid out in a randomized block design. Five of the replicates were laid out in a grid, with one replicate per row of trees. The remaining replicate was placed out in a 2 × 2 array. In addition to catches of *B*. *aquilonis*, there were large catches another fruit fly, *B*. *tenuifascia*, so the catch of these flies was also analysed.

Target species were caught only in traps that contained their specific lure (i.e. no *C*. *capitata* or *B*. *dorsalis* were trapped in cuelure-only traps). Consequently, analyses for the different species did not include the traps that did not contain their lure.

### Surveillance sensitivity

The probability of an insect being trapped in a trap is a function of distance from the trap and time, as well as biological attributes (e.g. sex, age, prior feeding) and abiotic habitat and climate variables. The probability of an insect being attracted to, and entering a trap, decreases as its original distance from the trap increases. If this is displayed as a catch probability surface, it will show a high probability of catch at the trap in the centre, with catch tailing off with increasing distance 360° around the trap (assuming no wind etc.), similar to the shape a ball under a blanket would make. The Effective Sampling Area (ESA) condenses the area under the probability surface into a cylinder of unit height, the area of the top of the resulting cylinder is the ESA. This value is not an estimate of the actual trapping area of a trap; rather, this value can be interpreted as a density conversion coefficient that can then be used to estimate the probability of capture per area over time^20,21^. The estimated daily ESA of cuelure traps for male *B*. *tryoni* from Stringer *et al*.^40^, was used to estimate the probability that a uniformly distributed *B*. *tryoni* would be trapped (Ptrapped) in a grid of traps following the inverse cube law for detection, eqn. 1^51,52^.

1 $${{\rm{P}}}_{{\rm{trapped}}}={\rm{ERF}}\times (\frac{\sqrt{\pi }}{2}\times {\rm{ESA}}\times {\rm{Trap}}\,{\rm{density}})={\rm{ERF}}\times (\frac{\sqrt{\pi }}{2}\times {\rm{Trap}}\,{\rm{cover}})$$

Trap cover is the combined area that is covered by the traps and their effective sampling areas; ERF denotes the Gauss error function^20,53^, or the probability that the $\frac{\surd \pi }{2}$ × Trap cover value encompasses the whole area to be sampled. As the $\frac{\surd \pi }{2}$ × Trap cover value increases, the probability that a fly will be trapped increases. Once P_trapped_ is estimated, the catch can be predicted by multiplying P_trapped_ × Pop (trappable population of males). Using this estimate as the target catch to determine the amount of change in the catch of flies in response to different lure combinations, the proportion of the catch of the target species in the multiple-lured traps was divided by the catch in its single-lured specific trap:

$${\rm{Proportion}}\,{\rm{of}}\,{\rm{catch}}={{{\rm{P}}}_{{\rm{catch}}}}^{\mbox{'}}=({\rm{catch}}\,{\rm{in}}\,{\rm{multiple}}\,-\,{\rm{lured}}\,\mathrm{trap}/\mathrm{catch}\,{\rm{in}}\,{\rm{single}}\,-\,{\rm{lured}}\,{\rm{trap}})$$

The inverse of Pcatch’ was calculated to assess how that reduction could be reversed (eqn. 2). The result was hypothesised to be the multiple by which the original trap density needed to be increased by to regain the trap sensitivity of the single-lure trap surveillance system.

2 $${\rm{I}}{\rm{n}}{\rm{c}}{\rm{r}}{\rm{e}}{\rm{a}}{\rm{s}}{\rm{e}}\,{\rm{i}}{\rm{n}}\,{\rm{t}}{\rm{r}}{\rm{a}}{\rm{p}}\,{\rm{d}}{\rm{e}}{\rm{n}}{\rm{s}}{\rm{i}}{\rm{t}}{\rm{y}}\,{\rm{r}}{\rm{e}}{\rm{q}}{\rm{u}}{\rm{i}}{\rm{r}}{\rm{e}}{\rm{d}}={{{\rm{N}}}_{{\rm{t}}{\rm{r}}{\rm{a}}{\rm{p}}{\rm{s}}}}^{\mbox{'}}={1/{{\rm{P}}}_{{\rm{c}}{\rm{a}}{\rm{t}}{\rm{c}}{\rm{h}}}}^{\mbox{'}}$$

We tested this hypothesis by calculating the number of flies from a hypothetical population of 80 trappable male *B*. *tryoni* expected to be captured in a single-lure trap surveillance system N_pop_. We then compared this with the expected catch in a multi-lure surveillance system N_pop_’ (eqn. 3) when there had been an increase in trap density (N_traps_’).

3 $${{{\rm{N}}}_{{\rm{p}}{\rm{o}}{\rm{p}}}}^{\mbox{'}}={\rm{P}}{\rm{o}}{\rm{p}}\times {{\rm{P}}}_{{\rm{t}}{\rm{r}}{\rm{a}}{\rm{p}}{\rm{p}}{\rm{e}}{\rm{d}}}\times {{{\rm{P}}}_{{\rm{c}}{\rm{a}}{\rm{t}}{\rm{c}}{\rm{h}}}}^{\mbox{'}}\times {{{\rm{N}}}_{{\rm{t}}{\rm{r}}{\rm{a}}{\rm{p}}{\rm{s}}}}^{\mbox{'}}$$

### Statistical Analyses

Analysis methods for the four trials were similar. To allow a measure of variability to be included in figures, data at each assessment were analysed using a Poisson generalized linear model (GLM^54^), with a logarithmic link. Where there was over-dispersion (dispersion >1), this was estimated.

Total counts for each trap were calculated, and analysed formally. Initially, the total counts were analysed with a Poisson-gamma Hierarchical Generalized Linear Model (HGLM)^55^, which included lure as a fixed effect with Poisson distribution and logarithmic link, and spatial factors (replicate etc.) as random effects with a gamma distribution and logarithmic link. The importance of the spatial factors was assessed by a Χ^2^ test of the change in deviance on dropping the term, as implemented in GenStat’s HGRTEST procedure (GenStat Committee 2015). Where random effects were found to be important, the fixed treatment effects, and any contrasts between treatments, were assessed using a similar test as implemented in GenStat’s HGFTEST procedure, but using an F-test of the statistic to adjust for bias in a similar manner to that used for REML^56^. Denominator degrees of freedom for this were those associated with traps.

Where Poisson GLM were used for the final analyses (when there we not any important random effects), treatments were assessed using F-tests where there was over-dispersion, and Χ^2^ tests otherwise.

In the results, approximate 95% confidence limits were obtained on the logarithmic scale, and back-transformed for presentation. In the figures illustrating changes with assessment, all results were adjusted to flies/trap/day for the assessment periods and catch per trap over then entire trial. For clarity, confidence limits for only three selected means from within the plot are shown, but drawn to the side.

All analyses were carried out with GenStat v. 18^57^.

### Compliance with ethical standards

This article does not contain any studies with human participants performed by any of the authors. All applicable international, national, and/or institutional guidelines for the care and use of animals were followed.

## Supplementary information

Supplementary File

## Data Availability

Data are stored at The New Zealand Institute for Plant & Food Research Ltd. and are available from the lead author on request.
