# Supplementary material for: Effect of Lure Combination on Fruit Fly Surveillance Sensitivity
Source: Sci Rep. 2019 Feb 25;9:2653. doi: 10.1038/s41598-018-37487-6 (PMC6390102; doi:10.1038/s41598-018-37487-6)
Supplement: Supplementary file 1 — Supplementary File [file 41598_2018_37487_MOESM1_ESM.docx]

Title: Effect of Lure Combination on Fruit Fly Surveillance Sensitivity

Authors: Lloyd D. Stringer, Rajendra Soopaya, Ruth C. Butler, Roger I. Vargas, Steven K. Souder, Andrew J. Jessup, Bill Woods, Peter J. Cook and David Maxwell Suckling

**Whānui (manuscript abstract in Te Reo- Māori)**

He nui te utu mo te tirotiro mo nga orotā pepeke, a kaore i kitea e te kaihokohoko he orotā whāinga.

Mai i te whakaturanga angitu orotā o mua he takahanga rereke, he maha nga mōunu whakanohoia ki tetahi rore kotahi, kia piki ake te kakamatanga o te pūnaha tirotiro.

I tirohia e matou te whai hua o te tūhononga o te mōunu mo nga *Tephritidae*- te *trimedlure, cuelure, raspberry ketone* me te *methyl eugenol*- I runga I nga parakete o *Ceratitis capitata, Zeugodacus cucurbitae, Bactrocera tryoni, B. dorsalis, B. aquilonis* me te *B. tenuifascia* i roto i a Ahitareiria me Amerika (kaore nga momo katoa kei roto i ia whenua)*.*

Tuhinga o mua te mātotorutanga o nga rore e hiahia ana hei whakaheke i te whakaiti o te parakete nā te aroaro o nga mōunu mo nga Tephritidae kua āta tatū.

Te meatanga o te tuhinga o mua te mātotorutanga o nga rore kia mau tonu te tūtohutanga o te tirotiro kua whakatauira mo te taupori whakaaringa whakaaro o nga *B. tryoni* tane, me te waahi kohinga o nga rore *cuelure* mo ratou kua āta tatū.

Te tūhononga o nga mōunu e toru ka whakaitihia nui te parakete o te orotā he aroturuki ki te *methyl eugenol*, ko te *B. dorsalis.*

Ki to tatou ohoreretanga, i kitea e matou he rore *trimedlure* i kawe *methyl eugenol* ka mau ki te whakanuia I te 3.1 iti iho o te *C. capitata* whakaritea ki te rore trimedlure anake i Ahitereiria, engari i roto i Hawaii kahore he rereketanga i nga parakete i kitea.

I runga i te raraunga i konei me te rangahau i whai mai, te tūhononga o etahi mōunu tane mo te kimi moata o nga ngaro *tephritid* ka puta te hototahi, mehemea, he whakaheke i te tūtohutanga o te tirotiro kua kitea, ka taea te whakaheke ma te whakanui ake te mātotorutanga o nga rore i roto i te waahi.
